# Evolution of the Fatigue Failure Prediction Process from Experiment to Artificial Intelligence: A Review

**DOI:** 10.3390/ma18051153

**Published:** 2025-03-04

**Authors:** Cornel Samoila, Doru Ursutiu, Iuliana Tudorache (Nistor)

**Affiliations:** 1 Faculty of Material Science and Engineering, Transylvania University of Brasov, Bdul. Eroilor Nr. 29, 500036 Brasov, Romania; juliana.nistor@liebherr.com; 2 Technical Science Academy of Romania, Bd. Dacia Nr. 26, Sector 1, 010413 Bucharest, Romania; 3 Faculty of Electrical Engineering and Computer Science, Transylvania University of Brasov, Bdul. Eroilor Nr. 29, 500036 Brasov, Romania; ursutiu@unitbv.ro; 4 Academy of Romanian Scientists, Str. Ilfov Nr. 3, Sector 5, 030167 Bucharest, Romania

**Keywords:** failure, prediction, S-N curves, machine learning, deep learning, neural networks, hybrid fatigue models

## Abstract

An analysis of the time evolution of fatigue break prediction shows increasingly shorter developmental stages. The experimental period was the longest; the combination of more powerful mathematical methods led to a leap in evolution and a shortening of implementation time. All fatigue rupture prediction methods have proven to have limitations due to the multitude of influencing factors and the insufficient number of practical factors considered. Recently, attempts have been made to increase prediction accuracy by combining methods based on the physical mechanisms of the fatigue failure process with data-driven methods assisted by artificial intelligence. We attempt to present this evolution herein. There are several methods of review suitable for analyzing this subject: systematic, semi-systematic, and integrative. From these, a combination of semi-systematic and integrative was chosen precisely because the two methods complement each other.

## 1. Introduction

Since the beginning of fatigue testing, the prediction of the moment of rupture has been an important research objective. The field has developed such that, for a given steel in a given state of treatment, repetitive experimentation to determine the number of cycles to rupture is no longer necessary; instead, the moment of rupture can be estimated from a set of experiments considered as a model. Considering the multitude of experimental data in the field as well as the diversity of methods used for prediction, a classification of the methods used so far can be made (Table 1).

The first three methods are based on the physical mechanisms of fatigue rupture process, while the last one is based on experimental data and their correlations according to AI methods.

Since fatigue approach methods (S-N, Murakami model, Coffin model, Basquin equation) and fracture mechanics (Irwin, etc.) describe the material–external force relationship at the macroscopic level without being very precise, the attention of researchers was directed to consider more influential factors. Thus, research began to address the structural defects representing important causes that determine material fatigue behavior [1]. Therefore, Murakami predicted fatigue limit by considering mesoscopic defects and proposed addressing fatigue failure by considering the localization and size of defects. The Murakami model was proposed to predict the fatigue limit of small-size defects (<1000 μ m). In addition to Murakami’s considerations, Îtziar and later Li introduced the stress concentration coefficient and later combined this coefficient with the strain parameter. Encouraged by the increase in the quality of predictions, Mass-Williams et al. and Samuel et al. proposed considering the relative stress intensity factor by carefully taking into account the location, size, morphology, and interaction of defects.

Returning to Murakami, his model considers the arrangement of defects on the free surface as follows [2]:

- For surface defects,

(1) $$\sigma_{w}=1.43\cdot \left(H_{V}+120\right)\cdot {\left(\sqrt{{area}_{max}}\right)}^{\frac{1}{6}}$$

- For defects located just below the surface,

(2) $$\sigma_{w}=1.41\cdot \left(H_{V}+120\right)\cdot {\left(\sqrt{{area}_{max}}\right)}^{\frac{1}{6}}$$

- For the internal defects,

(3) $$\sigma_{w}=1.56\cdot \left(H_{V}+120\right)\cdot {\left(\sqrt{{area}_{max}}\right)}^{\frac{1}{6}}$$

where the following definitions hold:

$H_{V}$—the Vickers hardness [Kgf/mm^2^];
$\sqrt{{area}_{max}}$ is the maximum expected size of inclusions contained in a volume.

For the voltage intensity factor ($∆K_{th}$), Murakami proposes a more complex calculation formula not only related to hardness:

(4) $$∆K_{th}=C_{1}\cdot \left(H_{V}+C_{2}\right)\cdot {\left(\sqrt{{area}_{max}}\right)}^{\frac{1}{3}}$$

where the following definitions hold:

$C_{1}$—a material-independent constant with value $C_{1}$= 3.3 × 10^−3^;
$C_{2}$—a material-independent constant with value $C_{2}$ = 120.

Murakami and Endo [3] and Murakami [4] proposed a method for predicting the upper and lower limits of fatigue endurance. The upper limit of fatigue endurance corresponds to *σ_wu_* when defects or inclusions do not affect the fatigue fracture, and its value can be empirically estimated as follows [5]:

(5) $$\sigma_{wu}=1.6\cdot H_{v}+0.1\cdot H_{V}\;\mathrm{when}\;H_{V}<400$$

A lower limit *σ_wl_* of fatigue resistance can be obtained when a large inclusion is located in contact with the surface of a specimen [5]. The prediction equation is

(6) $$\sigma_{\mathrm{wl}}=1\text{:}41.(HV+120)/{\sqrt{{area}_{max}}}^{1/6}$$

To estimate $\sqrt{{area}_{max}}$, Murakami et al. [5,6,7,8,9] applied the extreme value statistic to the distribution of inclusions. Murakami suggested that, in terms of fatigue limit, a flaw could be a crack equivalent, so they established an empirical relationship between fatigue limit and flaw size utilizing the surface area parameter:

(7) $$\sigma_{w}^{n}\cdot \sqrt{area}=C_{n}\approx 6$$

where the following definitions hold:

$\sigma_{w}^{n}$ is the fatigue limit in rotational bending or tensile compression;
$\sqrt{area}$—the projection of the fault in the plane perpendicular to the direction of the maximum main stress;
C—a constant of the material.

In 1986, Murakami and Endo [2,3] proposed an empirical equation in which the fatigue limit, as well as the threshold of the stress intensity factor, are given as a function of the material hardness and the surface defect area:

(8) $${∆K}_{th}={3.31\times 10}^{-3}\cdot \left(H_{V}+120\right)\cdot {\left(\sqrt{area}\right)}^{1/3}$$

(9) $$\sigma_{w}=\frac{1.43\cdot \left(H_{V}+120\right)}{{\left(\sqrt{area}\right)}^{1/6}}$$

The appearance of hardness in Murakami’s equations can be explained by the fact that hardness is related to the plasticity of the metal mass under test, which the fatigue itself does not consider, being more related to the surface preparation.

To this brief presentation of Murakami’s theory and its extensions over time, it must be added that the dependence of material fatigue on material structure (phase transformations, sliding of grain boundaries, pore nucleation, micro-crack formation, structural defects, inhomogeneities, non-metallic inclusions, and chemical composition) is extremely important because traditional theories, of fracture mechanics, are based on concentration factors produced by structural defects [9]. As stated above, Murakami’s theory applies to defects larger than 1000 μm. Below this limit, the errors increase with decreasing size, to the point of rendering the theories inefficient, and the good convergence with existing experiments above the mentioned limit becomes almost nil. Going further in the field of structural dependence, we should also review the contribution of Zhu et al. who propose a new parameter, Z, to predict fatigue life, considering both the size and the location of inclusions [10,11,12,13]. In [14], the author proposed a model for predicting the VHCF (very high cycle fatigue life) regime > 10^7^ cycles by modifying the Murakami model [10,14], as shown in Equation (10),

(10) $${C=\left\{\sigma_{a}\cdot {\left(area\right)}^{1/12}\cdot D^{\beta }\right\}}^{\alpha }\cdot N_{f}$$

and a life-control parameter, as shown in Equation (11),

(11) $$Z=\sigma_{a}\cdot {\left(area\right)}^{1/12}\cdot D^{\beta }$$

where the following definitions hold:

$\sigma_{a}$ is the stress amplitude in MPa.
$\left(area\right)$ is the critical dimension of inclusion in m^2^.
α and *C* are the adjusted material parameters.
β is a material-dependent constant.
*N_f_* > 10^7^ cycles.
$D=\frac{d-d_{inc}}{d}$—the relative depth of the critical inclusion (*d* being the diameter of the minimum cross-sectional area of the specimen and *d_inc_* being the micro-defect size).

The relationship between the new parameter *Z* and fatigue life is very close to the experimental re-alignment. The parameter *D* in the modified life control model can express the inhomogeneous stress distribution in the cross-section and the influence of residual stress [15], which is a step forward from the original Murakami model [2], in which only the global stress was represented.

An interesting addition to the VHCF idea is found in the work of Tanaka and Akiniwa [8], who consider the power-law dependence of the stress intensity range and crack propagation rate, σa/∆N, inside the facet, as shown in Equation (12).

(12) $$\frac{\sigma_{a}}{∆N}=C\cdot {∆K}^{n}$$

By integrating this equation, they approximate the number of cycles to failure, *N* using Equation (13):

(13) $$\frac{N}{\sqrt{{area}_{inc}}}C\cdot \frac{2}{C\cdot \left(n-2\right)}\cdot ∆K_{inc}$$

Experiments by Tanaka and Akiniwa [8] led to the values *n* = 14.5 and *C* = 4.86 × 10^−21^ or *n* = 14.2 and *C* = 3.44 × 10^−21^, very close to each other but also satisfactory for the experiment.

El Haddad et al. [15] suggested a theoretical description of the Kitagawa curve [16] by introducing a “critical length” describing the transition between the short and long crack regimes. El-Haddad used the threshold value of the stress intensity factor (SIF) to calculate the critical crack size. A defect larger than the critical value will exceed the SIF threshold and lead to failure, while for cracks smaller than the critical size, the SIF may not increase and lead to failure.

(14) $$∆\sigma =\frac{∆K_{th}}{Y\cdot {\left(\pi \left(a-a_{0}\right)\right)}^{1/2}}$$

where the following definitions hold:

∆σ is the fatigue stress of the material containing a crack.
$∆K_{th}$—the stress intensity factor.
Y—the SIF form factor (threshold voltage intensity factor).
a—the independent variable representing the length of a crack.
$a_{0}$—the El-Haddad parameter represents the critical crack length of the material.

This “critical length” a_0_ suggests the Kitagawa curve as [17] not being intrinsic to a given material but having a strong relationship with microstructure, especially through the grain dimension in polycrystalline metals.

The Kitagawa diagram shows the fatigue limit decreasing with increasing defect size. Comparing the slope of this curve with that obtained by working with Murakami’s theory, we observe that there are two slopes. Kitagawa’s slope is one-half, and Murakami’s is one-sixth. Murakami covers most of the experimental results on metallic materials well.

Taking into account the limitations of all known prediction methods, lately, attempts are being made to increase the prediction accuracy by combining methods based on the physical mechanisms of the fatigue rupture process with AI-assisted data-driven methods.

If one refers to the essence of the fatigue fracture process using the symmetric alternating cycle test, the stages of the fracture process are the same [18,19]:

- Discontinuous phase—when nanoscale cracks start to appear.
- Continuous phase—when short, micrometer-scale cracks are formed by the random joining of nanometer-scale cracks.
- Fast phase—when long cracks appear, and the threshold of rupture is imminent.

It is not possible to establish a law of crack growth as a function of applied stress because the phenomenon is random depending on the distribution (also random) of singularities, discontinuities, slip bands, pores, and inclusions in the tested sample specimen. The crack growth law accepted by most researchers is the Paris–Erdogan Law (https://ro.wikipedia.org/wiki/Legea_lui_Paris, accessed on 5 January 2025). The law applies to micrometric cracks, the size of which can be measured because the dependent variable in the equation is the crack length “l”. The number of cycles remaining until the final rupture is expressed using the Paris–Erdogan law by the relation (15):

(15) $$N_{c}=\frac{2\cdot \left(l_{c}^{\frac{2-m}{2}}-{\;l}_{i}^{\frac{2-m}{2}}\right)}{\left(2-m\right)\cdot C\cdot {\left(∆\sigma \cdot Z\cdot \sqrt{\pi }\right)}^{m}}$$

where the following definitions hold:

$N_{c}$ is the number of cycles until breakage.
$l_{c}$—crack length at the critical moment of rupture.
$l_{i}$—the length of the crack at the initial time of testing.
C, m—constants of material.
∆σ—the amplitude of cyclic stress.
Z—a dimensionless parameter.

**Figure 1:**
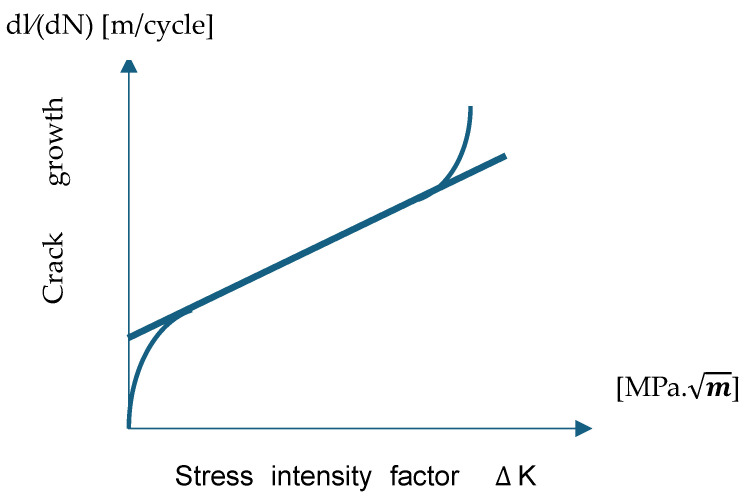
Correlation between the amplitude stress intensity factor and crack growth.

Most often, the dimensionless parameter Z depends on the length of the crack l, so solving the above equation requires numerical methods. The practical diagrams for the Paris–Erdogan Law are drawn in coordinates dl/dN representing the crack growth [m/cycle] and the stress intensity factor amplitude ∆K [MPa.$\sqrt{m}$] (with $∆K=K_{max}-K_{min}$), i.e., the difference between the maximum stress intensity factor and the minimum stress intensity factor (Figure 1). Table 2 summarizes known approaches for fatigue lifetimes.

## 2. Methods

The first stage of the work paper mode applied in the elaboration of the present paper was the definition of the aim. The aim was to trace the evolution of the approach to the problems of fatigue failure, especially those of the prediction of the moment of failure. The second stage was to list the criteria for the selection and inclusion of cited papers. The main criterion was the topic of the article that had to fit the purpose. From the plethora of papers that addressed the issue of fatigue burst prediction, those that contained turning points in the development of the topic were selected. This explains the rather large periods of the cited papers. This criterion also explains why the place of publication (book, ISI journal, BDI journal, or Proceedings) was not considered. It was important to mark the conceptual approach and not the place of publication. It will also be noted that an important criterion was to emphasize the expertise of the authors in the reviewed field, without, however, claiming that all recognized personalities in the field were mentioned. Too exhaustive an approach in terms of recognized authors would have made the reading too cumbersome, and the ultimate goal of highlighting developments would have been burdened with too much redundant information. Among the methods of analysis, systematic, semi-systematic, and integrative, a combination of semi-systematic and integrative was chosen precisely because the two approaches complement each other. The semi-systematic analysis allows one to establish a chronology of the topic addressed, while the integrative analysis is useful when the aim of the review is not to cover all the articles published on the topic under review, but to combine perspectives to highlight development patterns in the field.

The semi-systematic method was imposed by the fact that the problem of prediction of fatigue damage has been approached from different angles by different groups of researchers, coming from different disciplines (mechanics, materials science, resistance, solid state physics, etc.), which prevents a systematic review, since it is simply impossible to approach every single article dealing with the topic. In addition, a semi-systematic review allows revealing how the field has progressed over time, using meta-narratives instead of effect size measurement to do so (Wong et al., 2013). Since the semi-systematic method does not include a critical and synthetic evaluation, the authors also used an integrative approach that generates new perspectives (Torraco, 2005). In addition, this type of approach calls for more creative data collection, since the aim is not to cover all the articles published on the topic but to reveal perspectives. So, instead of being descriptive and historical, the authors preferred to try to reveal the new conceptual framework in which the prediction of burnout fatigue is beginning to develop with the advent of AI.

## 3. Results and Discussion

**Figure 2:**
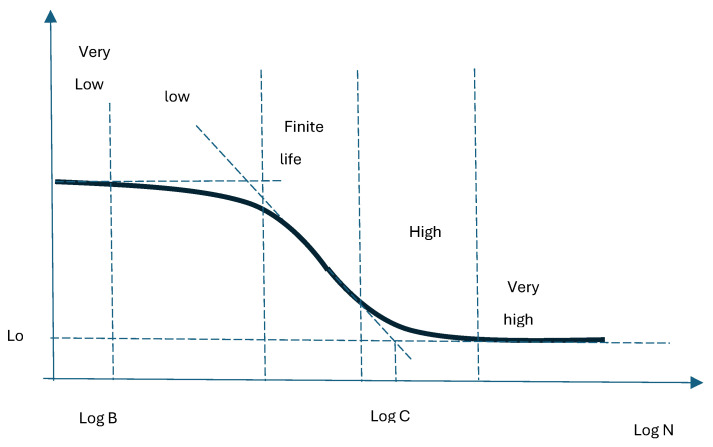
Schematic stress–life curve (Kohout–Vĕchet) [31].

The real difficulty in attempting to predict fatigue life is the diversity of stresses given. These can be static, quasi-static, dynamic cycling, or random dynamic. Hence, there is a diversity of parameters to be considered in predictions. Many papers consider a single parameter as the defining parameter and put the others at a secondary level, which simplifies the experiments but introduces a large inaccuracy factor. Among the parameters that are defined are the deformation parameter, the energy-based parameter, the SWT (Smith–Watson–Topper) parameter, and the Walker-like (also deformation) parameter. The consideration of these parameters is performed either for low cycle fatigue (LCF) or high cycle fatigue (HCF) fatigue regimes. A special situation that is worth presenting is the KV (Kohout–Vĕchet) fatigue model [31]. This model considers both the LCF and the HCF fatigue regimes. Mathematically, this union has the expression

(16) $$\sigma \left(N\right)=a{\left[\frac{\left(N+B\right)\cdot C}{N+C}\right]}^{b}\equiv \sigma_{\infty }\cdot {\left(\frac{N+B}{N+C}\right)}^{b}\equiv \sigma_{1}\cdot {\left(\frac{1+N/B}{1+N/C}\right)}^{b}$$

where the following definitions hold:

*n* is the number of cycles until the break.
*a* and *b* are the Basquin parameters.
$\sigma_{\infty }$—the fatigue limit.
$\sigma_{1}$—maximum tensile strength.

(17) $$B=\beta \cdot C\;\mathrm{cu}\;\beta ={\left(\frac{\sigma_{1}}{\sigma_{\infty }}\right)}^{1/b}$$

represents the number of cycles read at the intersection of the tangent of the region representing finite life with the horizontal asymptote of the maximum tensile strength (Figure 2) [31];

$$C=10^{7}\cdot \frac{1-\gamma }{\gamma -\beta }\;with\;\gamma ={\left(\frac{\sigma_{c}}{\sigma_{\infty }}\right)}^{1/b}\text{and}\;\sigma_{c}\;\text{resistance}\;\text{to}\;\text{fatigue}\;\text{for}\;10^{7}\;\text{cycles}$$

represents the number of cycles read at the intersection of the tangent of the region representing the finite lifetime with the horizontal asymptote of the fatigue limit (Figure 2) [31].

Karunananda [32] generalized the KV model by moving from considering stress to considering strain. The parameters considered (Table 3) are the Smith–Watson–Topper (SWT) parameter, the Walker-type (deformation) parameter (W), and an energy-based parameter (E) under uniaxial loading.

Before the generalized models based on the parameters in Table 3 are presented, the Kohout–Vĕchet fatigue model [36] for several parameters that determine fatigue damage, namely stress, strain, and energy, under uniaxial loading will be presented:

(18) $$\varphi \left(N\right)=:\varphi_{e}{\left(\frac{N+N_{u}}{N+N_{e}}\right)}^{b^{\prime }}\equiv \varphi^{ULCF}{\left[\frac{\left(N+N_{u}\right)\cdot N_{e}}{N+N_{e}}\right]}^{b^{\prime }}\equiv \varphi^{UHCF}{\left(\frac{1+N_{u}/N_{e}}{1+N/N_{e}}\right)}^{b^{\prime }}$$

where the following definitions hold:

$\varphi \left(N\right)$ is the parameter of fatigue damage.
$\varphi_{e}$—fatigue damage threshold parameter.
$\varphi^{ULCF}$—the fatigue impairment parameter for the low cycle fatigue regime.
$\varphi^{UHCF}$—the fatigue impairment parameter for the high cycle fatigue regime.
$N_{e}$—number of cycles to failure when strain is $\varepsilon_{e}$;
$N_{u}$—the number of cycles until the intersection of the tangent of the finite-life region with the horizontal asymptote of the elastic stress.
$b^{\prime }$ is the slope of the region of finite life.

How Equation (18) changes when considering the parameters from Table 3 is shown in Table 4.

In attempts to predict fatigue failure, it is important to consider several damage parameters. The KV model takes this approach by mathematically describing the combination of low- and high-stress cycles. In addition to this unification, the KV model considers deformation parameters as well as energy parameters, which widens the range of practical cases that can be addressed. This is essential when considering that metallic systems are loaded either quasi-statically (monotonic), dynamically, or dynamically–cyclically.

Another approach for predicting fatigue life is the statistical one. It starts from the observation that fatigue limit S_∞_ is a random variable considering the randomness of the structural distribution of inclusions and dislocations in the metallic structure. It has the mean *µ* and the variance *σ_2_*. If reference is also made to *N_f_* which symbolizes a finite lifetime, its logarithm is also a random variable with a normal distribution. The variance of the logarithm of finite life *ln* (*N_f_*) is *τ*_2_, and the logarithmic value of the stress S represents the variable x = ln(S) from waiting v(x) [36,37,38,39,40,41,42,43,44,45,46].

A likelihood function is defined, to be able to estimate the undeterminable parameters *L(θ)*. This function considers both censored C (specimen did not break) and uncensored U (specimen broke) attempts [38]:

(19) $$L\left(\theta \right)=\prod_{i\in U}f\left(y_{i};\left(\tau ,v\left(x_{i}\right)\right)\right)\cdot \emptyset \left(\frac{s_{i}-\mu }{\sigma }\right)\times \prod_{i\in C}\left(1-\emptyset \left(\frac{y_{ro}-v\left(x_{i}\right)}{\tau }\right)\cdot \emptyset \left(\frac{s_{i}-\mu }{\sigma }\right)\right)\;$$

where the following definitions hold:

f is the finite lifetime density function.
∅—cumulative distribution function for a standard normal variable.
$\tau^{2}$—variance of the logarithm of finite life.
$y_{ro}$—the value of observation.

From Equation (6), we derive the probability (P) that the specimens did not break (censored):

(20) $$P=1-\emptyset \left(\frac{y_{ro}-v\left(x_{i}\right)}{\tau }\right)\cdot \emptyset \left(\frac{s_{i}-\mu }{\sigma }\right)$$

If a stress level S is considered, Equation (19) can be used to calculate the probability that the test specimen will not survive a duration of $N_{x}=e^{y_{x}}$,

(21) $$q=\emptyset \left(\frac{y_{x}-v\left(x\right)}{\tau }\right)\cdot \emptyset \left(\frac{s_{i}-\mu }{\sigma }\right)$$

Equation (20) solved as a function of $y_{x}$ and holding the probability constant leads to the S-N curve, so the S-N diagrams derive the probability that the test specimen will break after a certain duration.

Another approach in prediction is to consider average stresses [47] because they have been found to influence fatigue life. This approach is usually employed for parts that are subjected to varying stresses, so it is necessary to introduce an average value as a parameter. It was also found that this method is useful for high fatigue cycles (HCF). The main theories are summarized in Table 5.

Also statistical is the approach that uses a nonlinear function (Stüssi) for modeling the S-N curves combined with the Weibull distribution. The procedure overcomes the limitations of the Stüssi method which consists of not considering failures and re-tests [50]. The Weibull distribution was used because it is known to be perfectly adapted to the analysis of life spans. A Weibull distribution with three parameters was used, $W=\left(a,b,c\right)$. The Stüssi function used was

(22) $$∆\sigma =\frac{R_{m}+\alpha \cdot N^{\beta }\cdot ∆\sigma_{\infty }}{1+\alpha \cdot N^{\beta }}$$

where the following definitions hold:

∆σ—stress range during the fatigue test.
$R_{m}$—maximum tensile strength.
*N*—the number of charge cycles to break or end of test.
$∆\sigma_{\infty }$—the fatigue limit.
α, β—geometrical parameters.

The geometric parameters are determined using two values, A and B, obtained by linear regression from the experimental fatigue test data, and are given by the following equations:

$$\alpha =e^{-B/A}$$

$$\beta =\frac{1}{A}$$

If you define the Stüssi function as a random variable, that is,

(23) $$x=∆\sigma -\frac{R_{m}+\alpha \cdot N^{\beta }\cdot ∆\sigma_{\infty }}{1+\alpha \cdot N^{\beta }}$$

and consider the Weibull distribution as

$$\begin{array}{c} F\left(xI\;a,b,c\right)=1-exp\left[-{\left(\frac{x-a}{b}\right)}^{c}\right]\;\text{with}\;x\geq a,\;a\in R\;\text{being the}\;\text{Weibull parameter of location,}\\ b>0\;\text{being the Weibull parameter of the scale, and}\;c>0\;\text{being the Weibull shape parameter,} \end{array}$$

the calculations lead to the probability of breakage:

(24) $$p=1-exp\left\{-{\left[\frac{∆\sigma -\frac{R_{m}+\alpha \cdot N^{\beta }\cdot ∆\sigma_{\infty }}{1+\alpha \cdot N^{\beta }}-a}{b}\right]}^{c}\right\}$$

The PWM method for evaluating parameters a, b, and c is used [51].

**Figure 3:**
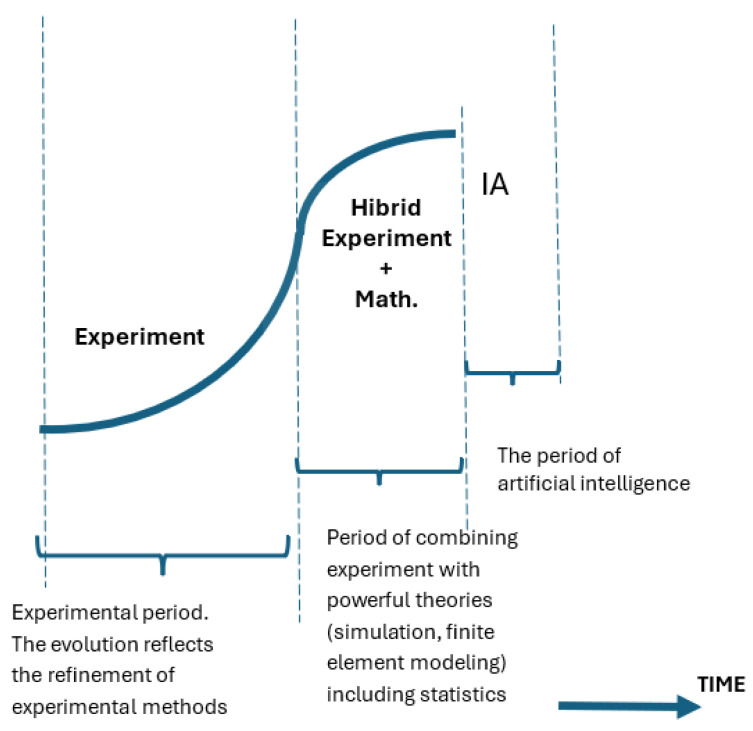
Evolution of the approach to fatigue failure prediction.

Compared to the period described so far, in which more or less accurate attempts were made to estimate the time of fatigue rupture, a rather strong tendency has recently emerged to solve this problem using the qualities of artificial intelligence (AI). The evolution of the approach to fatigue rupture prediction over time is shown in Figure 3.

First of all, the duration of the stages is becoming shorter and shorter. The longest period was the experimental one. The longest period was those based exclusively on experiments. Its combination with more and more powerful mathematical methods led to a leap in evolution and a shortening of the implementation time, which had begun to flatten out. The advent of AI brought about a new leap whose completion we cannot predict today. An overview of this method allows the following classification. In some cases, the S-N curves were too few to draw valid conclusions for breaking in fatigue. Some authors [52,53] have proposed the use of neural networks to train these sparse data and have drawn conclusions [54,55,56,57,58,59,60,61,62,63,64,65,66].

In a synoptic table of the years in which experimental works appeared, hybrid ones that combine experiments with mathematical and statistical modeling, and those that began to introduce AI, the result is a shortening of the durations between the nodal moments of evolution.

**Figure 4:**
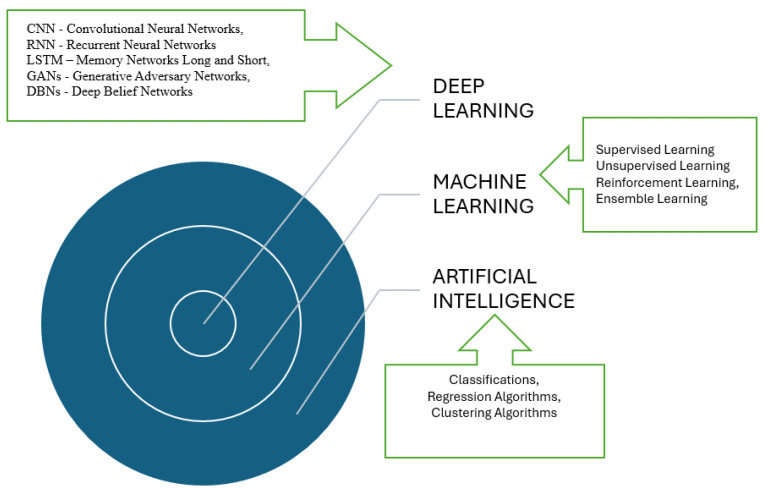
Basic components of AI, ML, and DL.

Before proceeding to the presentation of some applications using these techniques, an overview of systems belonging to AI is presented so that the reader understands the dependence and inclusion of methods that have begun to clarify or enable advanced processing of classical fatigue break data and break moment prediction (Figure 4).

The advent of AI has suggested to researchers in the field of fatigue rupture prediction that it can solve nonlinearity problems that classical methods have failed to address to a satisfactory extent. Table 6 presents a brief review of authors who have worked in the field of AI application in fracture mechanics and fatigue failure.

The main problem of using AI is to find a way to incorporate the laws governing fatigue rupture. The results of the application of AI have shown that it cannot substitute for an experiment but can only consider the nonlinearities introduced in the experiment by structural defects. AI without experimental data given by traditional methods is unusable. AI is now a system that enhances predictive capability in fatigue failure, even compensating for the lack of data. The most spectacular application of AI is in the field of maintenance, so it is directly linked to the reliability of the teams. AI in maintenance improves resource allocation and minimizes costs, thus increasing reliability and being applicable in many industries.

There are industrial applications of AI, especially in the field of maintenance. These applications use a few key technologies, among which are machine learning (decision trees, Support Vector Machines (SVMs), random forest, K-Means clustering, gradient boosting techniques such as XGBoost and LightGBM, and specialized models such as long short-term memory (LSTM)), deep learning (time series analysis, Recurrent Neural Networks (RNNs), Convolutional Neural Networks (CNNs), Computer Vision, etc. Manifestation forms of AI in the industry can be found in AI-based robotics, generative artificial intelligence models, blockchain technology, digital twins, explainable artificial intelligence, edge computing, and IoT integration. Entities that embrace this transformational concept will become highly competitive by showing that they understand that the industry will become increasingly dependent on data-driven decision-making [67].

It must be said that expert systems developed to solve fatigue break predictions have proven their limitations because they are oriented to an experimental specificity and therefore show a certain rigidity in their approach. AI broke these limitations, being more elastic and adaptable to situations that expert systems could not address correctly or had a large misjudgment. To predict the fatigue resistance of different steels, Agrawal [68] used neural networks, deciding trees, and multivariate polynomials as data processing tools. The final result was a more accurate evaluation than classical prediction methods.

It is presented in the following application of Artificial Neural Networks (ANNs) to problems of analyzing fatigue endurance data. In the first step, the experimental data are normalized (e.g., by logarithm) to be in the three layers of the input layer processed, the hidden layer, and the output layer. Six input variables are chosen, namely $\sigma_{TS}$, tensile stress; $\sigma_{YS}$, flow stress; $H_{V}$, hardness; $log\sigma_{a}$, the logarithm of the applied stress amplitude; and the reports $\sigma_{a}/\sigma_{TS}$, $\sigma_{a}/\sigma_{YS}$. The output variable for the analysis is logN, where *N* is the lifetime (number of cycles). To the input, variables are added to the chemical composition of the analyzed steel. All data together are quite a lot for analysis and can affect the accuracy of the ANN result. Therefore, the selection of the input variables is performed using Lasso (Last Absolute Shrinkage and Selection Operator), BIC (Bayesian Inference Criterion), and MAICE (Minimum Information Theoretical Criterion (AIC) Estimate). After their application, the input variables may be C%, Si%, Mn%, C%, $log\sigma_{a}$, $\sigma_{a}/\sigma_{TS}$, $\sigma_{a}/\sigma_{YS}$ if, for example, the steel is CAS6NM. By applying the random forest algorithm, the authors obtained “…*a slight over-fitting*…” which they consider acceptable. They did not determine a fatigue-breaking limit but considered the value of the input parameters at which the samples withstand 10^7^ cycles. In the mentioned study [69], a program called “Shiny Materials Genome Integration System for Phase and Property Analysis” (Shiny-MIPHA), developed by Adachi Laboratory [70], was used.

Another application of neural networks to fatigue fracture analysis is to use experimentally raised S-N curves to generalize their partial and specific conclusions to the whole class of steels [71]. We start from the data provided by the S-N curves using Equation (25):

(25) $$log\sigma_{a}=A-B{\left[logN\right]}^{p}$$

which is a kind of generalization of the power law, $\sigma_{a}$ being the alternating stress; the exponent *p* usually has a value of 1, and the constants A and B depend on each ratio of the tensile and compressive stresses. Before applying an ANN, a mathematical model is created. In the presented case, this model is realized by using a multilayer perceptron network having two neurons at the input for the used stress and the number of cycles and one neuron at the output for the alternative stress. The training was performed with the backpropagation algorithm based on the moment rule and the data provided by the S-N curves; the final conditioning being provided by Equation (26):

(26) $$\sigma_{a}=f\left(\sigma_{med},N\right)$$

where $\sigma_{med}$ is the average stress, $\left(\sigma_{max}+\sigma_{min}\right)/2$.

**Figure 5:**
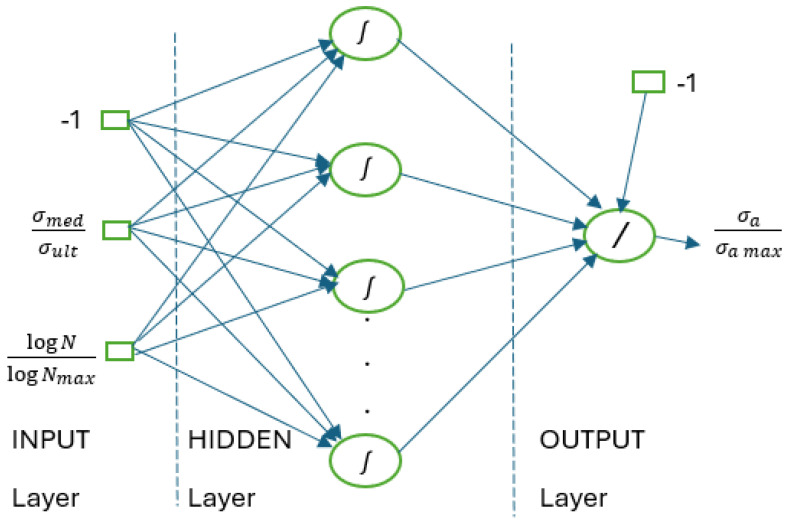
Simulation model using ANN [72].

In [72], the diagram of the simulation model used is also presented (Figure 5).

The limited driving conditions are ${\;N}_{max}=10^{7}$ cycles and $\sigma_{a\;max}=\frac{\sigma_{ult\;t\;}-\sigma_{ult\;c}}{2}$, and the definition of the two tensile and compressive stresses is $\sigma_{ult}=\sigma_{ult\;t}$ if $\sigma_{med}>o$ and $\sigma_{ult}=\sigma_{ult\;c}$ if $\sigma_{med}<o$, and R=−1. To increase the accuracy of the estimation, the learning is performed using several sets of values for R (which is the ratio between the minimum value of the stress and its maximum value, also taking into account the kind of tensile or compressive stress, the latter having a minus sign). Table 6 shows the values used in the presented work [71].

The four training sets recorded an increasingly smaller root mean square (RMS) error such that the 6R set yielded an error of 0.00015 and a correlation coefficient of 0.995. Thus, the application of the ANN method in the case of a small number of S-N curves leads to satisfactory results [72,73,74].

A variety of machine learning is Deep Learning, a system that uses more than one hidden layer of neural networks. The application of this method requires first avoiding errors caused by the different scaling of the measured quantities used in the computation. This is done by standardization which allows us to bring the sizes to a comparable form. The most used methods are Z-score normalization (standardization of the standard deviation), decimal scaling, and min/max scaling [75,76,77].

For standardization of the standard deviation, the relation (27) is used:

(27) $$Z=\frac{x-\mu }{\sigma }$$

where the following definitions hold:

x is the measured value of the size.
μ—average value of the size.
σ—standard deviation of the size.

**Figure 6:**
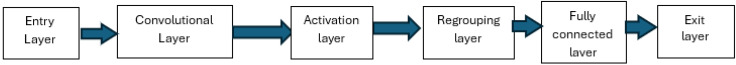
Layers in CNN networks.

In the cited work, the prediction was made using several input variables and only one output variable, which was a regression problem. The method used was CNN-LSTM (Convolutional Neural Network–Long Short-Term Memory Network). CNN networks are made up of several layers (Figure 6).

**Figure 7:**
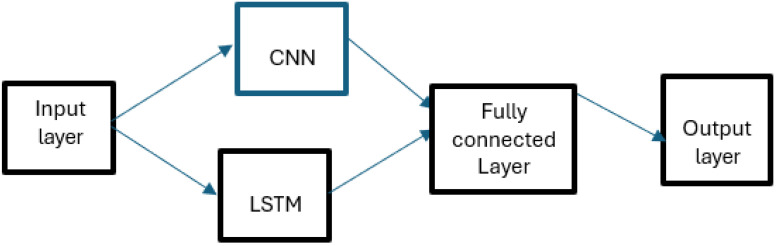
Structure of the CNN-LSTM model.

LSTM networks add and remove cellular states using a three-gate structure consisting of a forgetting gate, an entry gate, and an exit gate. The structure of this combined model is shown in Figure 7. 

After structuring, the CNN-LSTM model is trained by setting the optimization intervals of the parameters in the convolute layer, the LSTM layer, and the fully connected layer in advance (Table 7). Working parameters of the CNN-LSTM model (Table 8).

A comparison made with other models (RNN—Recurrent Neural Network, GRU—Gated Recurrent Unit, Transformer, and LeNet5—(Learnable Network with five parameter layers), shows that the CNN-LSTM model improves R2 (*coefficient of determination*) by up to 1.4%, reduces RMSE (*root mean square error*) by up to 14.9% and reduces MAPE (*mean absolute percentage error*) by up to 39%; thus, it has a better ability to predict fatigue endurance.

The application of AI in the field of fatigue burst prediction should not be seen as a panacea. This approach greatly helps to generalize and increase the accuracy of the results but cannot exclude the experimental part. Only experimentation provides reliable and accurate input measures. To complement the knowledge on the use of AI in the wider field of failure of steel parts and structures, a small summary is presented in Table 9 [76,78,79].

## 4. Conclusions

The analysis leads to the following conclusions:

1. AI is an important leap in the approach to steel fatigue and lifetime determination. It allows for reducing experimentation time and for obtaining viable conclusions from a smaller number of experiments.
2. AI is not a substitute for the known physical methods in the field of fatigue, but it increases the confidence in the results obtained by having a higher generalizability than all the methods tried so far.
3. The application of AI requires the mastery of software that fatigue testing laboratories have not yet generalized, so it will be some time before the methods for measuring and interpreting data obtained based on AI can be standardized.
4. The emergence of AI does not rule out experiments. It remains the basic linchpin of fatigue testing, so the endeavor to innovate in experiments must continue.
5. The main problem in using AI is to find a way to incorporate the laws that govern fatigue failure into it.
6. The results of the application of AI have shown that it cannot substitute for an experiment but can only consider the nonlinearities introduced in an experiment by structural defects.
7. The application of AI to fatigue rupture prediction in future research requires the establishment of a universally accepted standard. Today, each AI user has his or her own opinion about the variables to be considered. The industry needs certain and reproducible criteria.

The present review is intended as an indemnity to attempt standardization for fatigue lifetime prediction in steels by AI methods (ML and DL).

## Figures and Tables

**Table 1:** Life prediction pathways.

Nr. Crt.	Forecast Path	Details
1	The inductive method	Experiments were generalized inductively, with approximately valid conclusions for a whole class of steel. Empirical equations were used, but these were limited by idealizations and phenomenological assumptions, which affected their accuracy in relation to real phenomena.
2	The deductive method	From the experiments, general theories were deduced, with a greater or lesser degree of validity and accuracy in estimating the moment of breaking.
3	The simulation method	Using powerful software, experimental conditions and important process parameters can be simulated, and predictions have been attempted. The simulations, often based on finite elements, are limited by the reliability of the model and cannot capture variational laws. The simplifications that are resorted to give rise to uncertainties.
4	The use of Artificial Intelligence (AI)	Starting from big data processing, predictions can be made using artificial intelligence methods, the most widely used of which is machine learning. The biggest flaw of the machine learning method is that it is a kind of “black box” whose correlations and interferences drawn from the processed data are not based on physical interpretation.

**Table 2:** Lifetime approaches to fatigue.

Nr. Crt.	The Approach	Comment	Formulas
1	Applied stress $\sigma_{a}$–fatigue life $N_{f}\;$ [20]	It is the most commonly used in papers, generating the so-called S-N curves, where S is the test stress symbol and N is the fatigue life expressed in several cycles. Basquin established the formula, which is useful when the tests are in the elastic range.	$\sigma_{a}$ = $\;\frac{E\cdot ∆\varepsilon }{2}=\sigma_{f\cdot }{\left(2\cdot N_{f}\right)}^{b}$ E—Young’s modulus. ∆ε—range of elastic deformation. $\sigma_{f}$—fatigue resistance coefficient. b—the exponent of fatigue resistance.
2	Deformation–fatigue life [21]	The method considers both elasticity and plasticity. The Manson–Coffin equation.	$\frac{{∆\varepsilon }_{p}}{2}=\varepsilon_{f}^{\prime }\cdot {\left({2\cdot N}_{f}\right)}^{c}$ ${\;\;\varepsilon }_{f}^{\prime }$—ductile breakage coefficient. c—the exponent of ductile breakup.
3	Field intensity [22]	It is mainly used for notched parts and considers the stress distribution in the notches, which leads to the formation of fatigue cracks.	$\sigma_{F}=\frac{1}{V}\int_{\omega }^{1}f\left(\sigma_{ij}\right)\cdot \varphi \left(\underset{r}{\to }\right)\cdot dV$ ω—the local region of deterioration. $f\left(\sigma_{ij}\right)$—the deterioration function. $\varphi \left(\underset{r}{\to }\right)$—the weight function. *V*—the volume of the region ω.
4	The micro approach [23]	It refers to the motion of slip bands that accumulate energy due to the obstruction of their motion, by granular boundaries, and thus generate a distortion.	Represents the accumulation of plastic deformation stress in the slip band. ${∆}_{yps}\cdot N=C_{1}$ $\frac{∆\varepsilon_{p}}{M}\cdot \left(\frac{h}{w}\right)\cdot N=C_{1}$ h—the width of the slip band. w - distance. $∆\varepsilon_{p}$—the plastic deformation range. M—the Taylor factor.
5	The critical plane [24]	The Fatemi Society (FS) model is applied to multi-axial shear fatigue failure considering the shear strain amplitude.	$\frac{\tau_{max}}{\tau_{f}^{\prime }}\cdot \frac{∆\gamma }{2}+\frac{\sigma_{n,max}}{\sigma_{f}^{\prime }}\cdot \frac{∆\varepsilon }{2}=\frac{\tau_{f}^{\prime }}{G}\cdot {\left(2N_{f}\right)}^{2b}+\gamma_{f}^{\prime }\cdot {\left(2N_{f}\right)}^{c}$ ${\;\tau }_{max}$, $\sigma_{n,max}$ represent the maximum shear stresses and normal stresses, respectively. $\frac{\;\;∆\gamma }{\;2}$, $\frac{∆\varepsilon }{2}$—shear strain and normal strain amplitude. ${\;\;\tau }_{f}^{\prime }$, $\gamma_{f}^{\prime }$—shear fatigue and ductile shear strength. G—the shear modulus.
6	The energy approach [25,26,27,28,29,30]	Fatigue is the absorption and accumulation of energy. When the accumulation reaches a critical value, breakdown occurs. The equation describes the energy–life curve.	$W_{p}=D_{o}+D_{1}\cdot N=\int \sigma \cdot d\varepsilon$ ${\;\;W}_{p}$ is the energy of cyclic hysteresis, ${\;\;D}_{o}$, $D_{1}$—constants.
7	The mechanics of continuous damage [31]	Fatigue fracture is analyzed with defect formation and propagation. Nonlinear damage evolves by changing the load-carrying capacity.	$D=1-{\left[1-r^{1/\left(1-\alpha \right)}\right]}^{1/\left(1-\beta \right)}$ α is the function of the tension state. β—a constant. r—the state of determination.

**Table 3:** Deformation parameters.

Calculation Formula	The Meaning of Terms
*Smith–Watson–Topper (SWT)* [33] ${\;\sigma }_{m}\cdot \varepsilon_{a}=SWT={\left(\sigma_{f}^{\prime }\right)}^{2}\cdot \frac{{\left(2\cdot N_{f}\right)}^{2b}}{E}+\sigma_{f}^{\prime }\cdot \varepsilon_{f}^{\prime }\cdot {\left(2\cdot N_{f}\right)}^{b+c}$	$\sigma_{m}$—the maximum cycle stress. $\varepsilon_{a}$—the total deformation amplitude. $\sigma_{f}^{\prime }$—the fatigue resistance $N_{f}$—the number of cycles to failure. $\varepsilon_{f}^{\prime }$—the limit strain for the low-cycle fatigue regime.
*Walker-like strain* [34] $\varepsilon_{a}{\left(\frac{2}{1-R}\right)}^{1-\gamma }=\frac{\sigma_{fw}^{\prime }}{E}\cdot {\left(2N_{f}\right)}^{b_{w}}+\varepsilon_{fw}^{\prime }\cdot {\left(2N_{f}\right)}^{c_{w}}\cdot {\left(\frac{1-R}{2}\right)}^{\left(c_{w}\cdot b_{w}-1\right)\left(1-\gamma \right)}$	R—the stress ratio. $\sigma_{fw}^{\prime },\;b_{w},c_{w}$—the material parameters. γ—Walker constant adjustment. E—the modulus of elasticity.
*Energy-based* [35] $∆W_{t}=∆W_{e}^{+}+∆W_{p}=\frac{1}{2\cdot E}{\left(\frac{∆\sigma }{2}+\sigma_{m}\right)}^{2}+\frac{1-n^{\prime }}{1+n^{\prime }}\cdot ∆\sigma \cdot ∆\varepsilon^{p}$	∆σ—the stress range. $n^{\prime }$—the cyclic strain-hardening exponent. $∆W_{t}$—the total deformation energy range. $∆W_{e}^{+}$—the range of elastic strain energy. $∆W_{p}$—the plastic strain energy range. $∆\varepsilon^{p}$—the range of plastic deformation.

**Table 4:** Generalized KV model using deformation parameters.

Calculation Formula	The Meaning of Terms
*Walker-like strain* [31] $\varepsilon_{w}\left(N\right)=\varepsilon_{w,e}{\left(\frac{1+N_{u}}{1+N_{e}}\right)}^{b^{\prime }}\equiv \varepsilon_{w}^{ULCF}{\left[\frac{\left(N+N_{u}\right)N_{e}}{N+N_{e}}\right]}^{b^{\prime }}\equiv \varepsilon_{w}^{UHCF}{\left(\frac{1+N/N_{u}}{1+N/N_{e}}\right)}^{b^{\prime }}$	$\varepsilon_{w}\left(N\right)$—Walker-type deformation parameter. $\varepsilon_{w}^{ULCF}$—Walker-type final plastic strain amplitude for the low-cycle fatigue regime. $\varepsilon_{w}^{UHCF}$—Walker-type final plastic strain amplitude for the high-cycle fatigue regime.
*Smith–Watson–Topper (SWT)* [31] $SWT\left(N\right)={SWT}_{e}{\left(\frac{1+N_{u}}{1+N_{e}}\right)}^{b^{\prime }}\equiv {SWT}^{ULCF}{\left[\frac{\left(N+N_{u}\right)N_{e}}{N+N_{e}}\right]}^{b^{\prime }}\;\;\equiv {SWT}^{UHCF}{\left(\frac{1+N/N_{u}}{1+N/N_{e}}\right)}^{b^{\prime }}$	$SWT\left(N\right)$—fatigue damage parameter SWT. ${SWT}_{e}$—the fatigue damage limit parameter. ${SWT}^{ULCF}$—short cycle fatigue damage parameter. ${SWT}^{UHCF}$—high cycle fatigue damage parameter.
*Energy-based* [31] ${∆W}_{t}\left(N\right)={∆W}_{e}{\left(\frac{1+N_{u}}{1+N_{e}}\right)}^{b^{\prime }}\equiv {∆W}^{ULCF}{\left[\frac{\left(N+N_{u}\right)N_{e}}{N+N_{e}}\right]}^{b^{\prime }}\equiv {∆W}^{UHCF}{\left(\frac{1+N/N_{u}}{1+N/N_{e}}\right)}^{b^{\prime }}$	${∆W}^{ULCF}$, ${∆W}^{UHCF}$—low cycle and high cycle fatigue energy parameters, respectively.

**Table 5:** Mean stress theories.

The Model	Hypotheses	Meaning of Terms
*Goodman* [47] $\sigma_{ar}=\frac{\sigma_{a}}{1-\sigma_{m}/\sigma_{u}}$	Expresses equivalent stress using the ultimate tensile strength of the material, the stress amplitude, and the average stress.	$\sigma_{u}$ is the final tensile strength. $\sigma_{m}$—the average stress. $\sigma_{a}$—the stress amplitude. $\sigma_{ar}$—the equivalent stress.
Morrow–Gerber [47] $\sigma_{ar}=\frac{\sigma_{a}}{1-\sigma_{m}/\sigma_{f}}$	Replaces the ultimate tensile strength $\sigma_{u}$ with breaking strength $\sigma_{f}$	$\sigma_{f}$—the true breaking strength.
Manson–Halford [48] $a=a_{0}+\left(a_{f}-a_{0}\right)\cdot {\left(\frac{n}{N}\right)}^{\frac{2}{3}N^{0.4}}$	Based on crack propagation theory.	$a_{0}$—the initial crack length. $a_{f}$—crack size during fatigue loading, considered 0.18 inches. n—number of cycles at which the crack reaches the value an underload S. *N*—material lifetime under load S.
SWT [47] $\sigma_{ar}=\sqrt{\sigma_{max}\cdot \sigma_{a}}$	Calculates the equivalent stress $\sigma_{ar}$ using maximum stress and stress amplitude.	$\sigma_{max}$—maximum stress.
Walker [48] $\sigma_{ar}=\sigma_{max}^{1-\gamma }\cdot \sigma_{a}^{\gamma }=\sigma_{max}{\left(\frac{1-R}{2}\right)}^{\gamma }$	The Walker model considers that the average stress is material dependent. It therefore introduces a parameter γ which varies from 0 to 1, with small values of the intervalue indicating that the material is more dependent on the average stress.	Like above
(GSE) Generalized energy damage parameter [49] $W_{gen}={\left(\tau_{max}\frac{∆\gamma^{e}}{2}+\frac{∆\tau }{2}\frac{∆\gamma^{p}}{2}+\sigma_{n,max}\frac{∆\varepsilon_{n}^{e}}{2}\;\;+\frac{∆\sigma_{n}}{2}\frac{∆\varepsilon_{n}^{e}}{2}\right)}_{max}$	It is a parameter that considers both the normal and the shear energy occurring in the experimental plane.	$\tau_{max}$—maximum shear stress. $\sigma_{n;max}$—normal maximum effort. $W_{gen}$—the strain energy parameter. $\tau_{max}\frac{∆\gamma^{e}}{2}$—elastic shear energy. $\frac{∆\tau }{2}\frac{∆\gamma^{p}}{2}$—shear energy in the plastic state. $\sigma_{n;max}\frac{∆\varepsilon_{n}^{e}}{2}$—normal elastic strain energy. $\frac{∆\sigma_{n}}{2}\frac{∆\varepsilon_{n}^{e}}{2}$—normal strain energy in the plastic state.

**Table 6:** A view of AI applied in the field of prediction.

Authors	Problem Solved	The Type of AI Utilized
Gong et al., 2017 [39]	Solving the small dataset problem to improve the accuracy of efficiency analysis.	Virtual sampling generation technology
Chen et al., 2021 [40]	Established a fatigue life prediction model by considering defects.	Support Vector Machine (SVR)
Ebid et al., 2022 [41]; Badra et al., 2022 [42]	Predicting the compressive and shear strength of a plate.	Artificial Neural Network (ANN)
Salem and Deifalla, 2022 [43]	Predicting the bending strength of plates (99% accuracy).	Integrated reinforcement shaft model
Jia et al., 2023 [44]	Predicting the relationship between average material stress and stress amplitude.	Deep neural network model
Sun et al., 2022 [45]	Predictive data augmentation method that generates high-quality samples based on the original data.	Generative adversarial network (GAN)
Li et al., 2022 [46]	Increasing the accuracy of the predictive model by extending the sparse dataset.	Monte Carlo simulation
Mishra și Molinaro, 2022 [47]	How neural networks are trained on supervised learning problems concerning the laws of physics.	Physics-based neural networks (PINNs)
Wang et al., 2023 [48]	Paris–Erdogan formula and the normalized S-N curve.	Physically guided machine learning frameworks

**Table 7:** Tension ratios used in learning.

Ratio used	3R	4R	5R	6R
	10	10	10	2
	−2	−2	−2	10
	0.1	0.1	−1	−2
		0.5	0.1	−1
			0.5	0.1
				0.5

**Table 8:** Working parameters of the CNN-LSTM model [77].

Parameters Working	Working Interval				The Optimal Values
Learning Rate	0.0001	0.001		0.01	0.001
Dropout Rate	0.1		0.5		0.2
Convolution Kernel Size	2		7		3
Number of Convolution Filters	8		128		64
Number of LSTM Units	32		160		64
Number of Fully Connected Layers	4		16		2

**Table 9:** Broadening the application field of AI methods.

Univoc Mono Systems	
**BN** Bayesian network	It consists of a combination of graph theory and the probability of relationships between network nodes.
**ANN** Artificial Neural Network	See above examples.
**GA** Genetic Algorithm	It has been successfully applied to damage detection in structures.
**FL** Fuzzy Logic	If sufficient experimental tests and measurements are available, FL can contribute to fault diagnosis.
**CBR** Case-Based Reasoning	It is a method that can reduce the dependence of failure analysis on extensive experimental information.
**Hybrid Systems**	
**ANN + GA + FL**	Real-time crack identification.
**GA + neuro-fuzzy (ANFIS)**	It was used to detect bearing faults. With this hybrid method, the average testing accuracy increased by about 60%.
**ANN + GA**	Allows detection, identification, and level of gear failure.
**CBR + GA**	It was used to identify faulty aeronautical components.

## Data Availability

The original contributions presented in the study are included in the article, further inquiries can be directed to the corresponding author.

## Abbreviations

Symbol	Explanation
LCF	Low Cycle Fatigue
HCF	High Cycle Fatigue
VHCF	Very High Cycle Fatigue
SIF	Stress Intensity Factor
KV	Kohout–Vĕchet Fatigue Model
AI	Artificial Intelligence
CNN	Convolutional Neural Network
RNN	Recurrent Neural Network
LSTM	Long Short-Term Memory
DBNs	Deep Belief Networks
ML	Machine Learning
SVM	Support Vector Machine
ANN	Artificial Neural Network
RMSE	Root Mean Square Error
MAPE	Mean Absolute Percentage Error
BN	Bayesian Algorithm
GA	Genetic Algorithm
FL	Fuzzy Logic
CBR	Case-Based Reasoning
SWT	Smith–Watson–Topper
